# Supplementary material for: NSCLC Cells Resistance to PI3K/mTOR Inhibitors Is Mediated by Delta-6 Fatty Acid Desaturase (FADS2)
Source: Cells. 2022 Nov 22;11(23):3719. doi: 10.3390/cells11233719 (PMC9736998; doi:10.3390/cells11233719)
Supplement: Supplementary file 1 [file cells-11-03719-s001.zip › Supplemental tables S3-S5.pdf]

**Supplemental Table S3**. Statistical significance of the dose-response curves reported in **Figure 1A**. Analysis was performed with two-way ANOVA and Bonferroni post-test for multiple comparisons. *p-value<0.05, **p-value<0.01, ***p-value<0.001, ****p-value<0.0001.

| | **BEZ-235 (nM)** | | | | | | | | |
|---|---|---|---|---|---|---|---|---|---|
| | **2** | **4** | **8** | **16** | **32** | **64** | **128** | **256** | **512** |
| **wt *vs* wt_bez** | ns | ns | ns | ns | ns | *** | *** | ** | ** |
| **wt *vs* G12C** | ns | ns | ns | ns | ns | ns | ns | ns | ns |
| **wt_bez *vs* G12C_bez** | ns | ns | ns | ns | ns | ns | ns | ns | ns |
| **G12C *vs* G12C_bez** | ns | * | ns | ns | * | *** | **** | **** | *** |

**Supplemental Table S4**. Statistical significance of the dose-response curves reported in **Supplementary Figure S1B**. Analysis was performed with two-way ANOVA and Bonferroni post-test for multiple comparisons. *p-value<0.05, **p-value<0.01, ***p-value<0.001, ****p-value<0.0001.

| | **BEZ-235 (nM)** | | | | | |
|---|---|---|---|---|---|---|
| | **5** | **10** | **25** | **50** | **100** | **200** |
| **wt *vs* wt_bez** | ** | *** | ** | ns | ** | * |
| **wt *vs* G12C** | ns | * | ns | ns | ns | ns |
| **wt_bez *vs* G12C_bez** | ns | ns | ns | ns | ns | ns |
| **G12C *vs* G12C_bez** | ns | * | ** | * | ns | ns |

**Supplemental Table S5**. Statistical significance of the dose-response curves reported in **Supplementary Figure S1C**. Analysis was performed with two-way ANOVA and Bonferroni post-test for multiple comparisons. *p-value<0.05, **p-value<0.01, ***p-value<0.001, ****p-value<0.0001.

| | **BEZ-235 (nM)** | | | | | |
|---|---|---|---|---|---|---|
| | **5** | **10** | **25** | **50** | **100** | **200** |
| **wt *vs* wt_bez** | ns | ** | ** | ** | ** | ** |
| **wt *vs* G12C** | ns | ns | ns | ns | ns | ns |
| **wt_bez *vs* G12C_bez** | ns | ns | ns | ns | ns | ns |
| **G12C *vs* G12C_bez** | ns | * | **** | *** | *** | ** |
