# Supplementary material for: NSCLC Cells Resistance to PI3K/mTOR Inhibitors Is Mediated by Delta-6 Fatty Acid Desaturase (FADS2)
Source: Cells. 2022 Nov 22;11(23):3719. doi: 10.3390/cells11233719 (PMC9736998; doi:10.3390/cells11233719)
Supplement: Supplementary file 1 [file cells-11-03719-s001.zip › Supplementary figure S1.pdf]

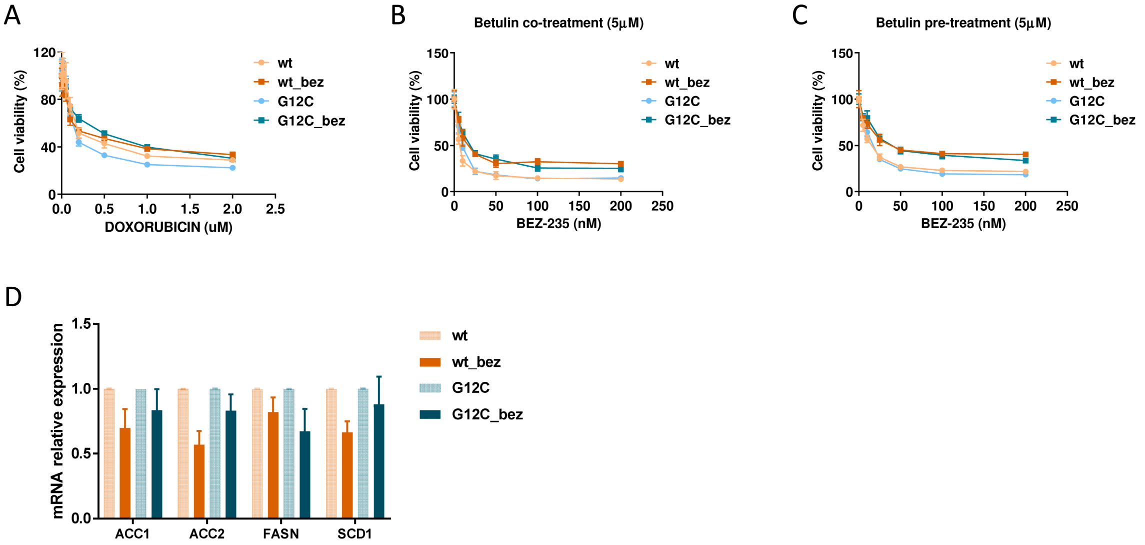

Figure S1. (**A**) Dose-response curves of the clones treated with doxorubicin at different concentrations. The mean and SD of at least three independent experiments are plotted. Statistical analysis was carried out through the two-way ANOVA test and the Bonferroni post hoc test for multiple comparisons, and any statistical significance was not detected. (**B**) Dose-response curves of the clones treated with increasing concentrations of BEZ235 in combination with betullin 5 uM. The means and SD of at least three independent experiments are plotted. Statistical analysis was carried out through the two-way ANOVA test and the Bonferroni post hoc test for multiple comparisons and is reported in Supplemental Table S4. (**C**) Dose-response curves of the clones pre-treated for seven days with betullin 5 uM, and then treated with increasing concentrations of BEZ235. The mean and SD of at least three independent experiments are plotted. Statistical analysis was carried out through the two-way ANOVA test and the Bonferroni post hoc test for multiple comparisons and is reported in Supplemental Table S5. (**D**) Histograms representing mRNA relative expression of the ACACA (ACC1 protein), ACACB (ACC2 protein), FASN, and SCD1. Actin was used as a housekeeping gene for normalization. The means of three replicates and SD are reported.
